# Role of ATP Hydrolysis in Cyanobacterial Circadian Oscillator

**DOI:** 10.1038/s41598-017-17717-z

**Published:** 2017-12-12

**Authors:** Sumita Das, Tomoki P. Terada, Masaki Sasai

**Affiliations:** 1 Department of Computational Science and Engineering, Nagoya University, Nagoya, 464-8603 Japan; 2 Department of Applied Physics, Nagoya University, Nagoya, 464-8603 Japan

## Abstract

A cyanobacterial protein KaiC shows a stable oscillation in its phosphorylation level with approximately one day period when three proteins, KaiA, KaiB, and KaiC, are incubated in the presence of ATP *in vitro*. During this oscillation, KaiC hydrolyzes more ATP molecules than required for phosphorylation. Here, in this report, a theoretical model of the KaiABC oscillator is developed to elucidate the role of this ATP consumption by assuming multifold feedback relations among reactions and structural transition in each KaiC molecule and the structure-dependent binding reactions among Kai proteins. Results of numerical simulation showed that ATP hydrolysis is a driving mechanism of the phosphorylation oscillation in the present model, and that the frequency of ATP hydrolysis in individual KaiC molecules is correlated to the frequency of oscillation in the ensemble of many Kai molecules, which indicates that the coherent oscillation is generated through the coupled microscopic intramolecular and ensemble-level many-molecular regulations.

## Introduction

**Figure 1:**
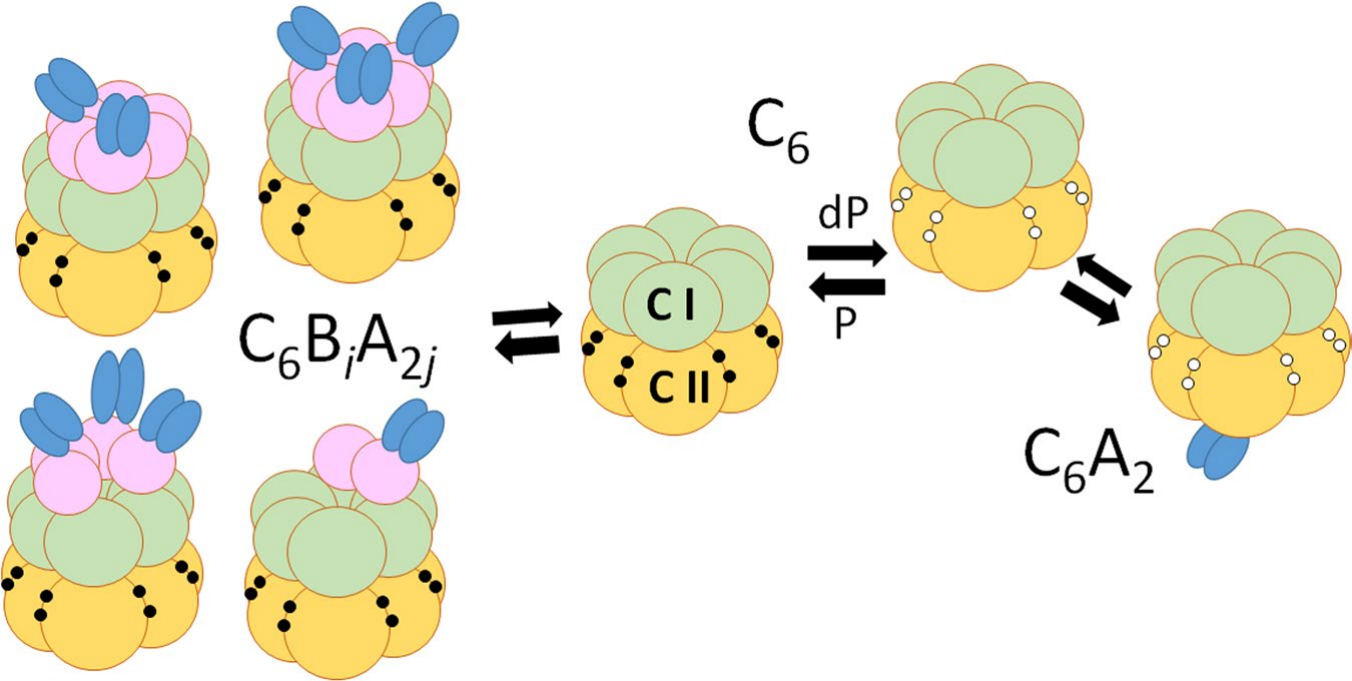
Scheme of interactions among Kai proteins. KaiC assembles into a hexamer C_6_ by forming CI-CII double rings. The CI ring (green) binds to KaiB (pink) to form C_6_B_*i*_ with 1 ≤ *i* ≤ 6 when the CII is phosphorylated (P), which further binds to KaiA dimers (blue) to form C_6_B_*i*_A_2*j*_ with 1 ≤ *j* ≤ *i* ^13^. The CII ring (yellow) binds to a KaiA dimer when dephosphorylated (dP) to form C_6_A_2_. Two specific sites, Ser431 and Thr432, in the CII of each subunit are phosphorylated (filled circle) or dephosphorylated (white circle). The CI domain exhibits slow ATPase reactions.

How is the coherent oscillation in biological system generated and how is it regulated by structures and reactions of constituent molecules? The system comprised of three cyanobacterial proteins, KaiA, KaiB, and KaiC, provides a unique opportunity to examine these problems^1–3^. As illustrated in Fig. 1, KaiC consists of tandemly repeated N-terminal (CI) and C-terminal (CII) domains^4^ and assembles into a hexamer^5,6^ by forming the CI-CII double rings^7^. By incubating KaiA, KaiB, and KaiC in the presence of ATP *in vitro*, two specific sites in the CII of each subunit of hexamer are repeatedly phosphorylated and dephosphorylated to exhibit a stable circadian oscillation^1,8,9^.

KaiA forms a dimer^10^ and binds to the CII ring of KaiC hexamer^11^ to promote the autophosphorylation of KaiC^12^. We write this KaiCA complex as C_6_A_2_. KaiB binds to the CI domain of each KaiC subunit to form C_6_B_6_^13^. Binding of KaiA to the CII is suppressed in C_6_B_6_, which promotes autodephosphorylation of KaiC^14,15^. Recent electron microscopic and mass spectrometric analyses^13^ showed that KaiB further binds to KaiA to form KaiCBA complex, C_6_B_6_A_2*j*_ with 1 ≤ *j* ≤ 6. When KaiA concentration is not too high, participation of KaiA in forming C_6_B_6_A_2*j*_ should sequestrate KaiA to suppress the C_6_A_2_ formation in the other KaiC hexamers, which may provide ensemble-level communication among KaiC hexamers. ATP molecules can bind on CI-CI and CII-CII interfaces of KaiC subunits; with slow ATPase activity, about 10 molecules of ATP are hydrolyzed per each CI domain and several ATP molecules per each CII domain in 24 hours^16,17^.

A striking feature is the strong correlation between the ATPase activity and the phosphorylation rhythm: When the KaiC sequence is mutated, the induced modulation of frequency of ATP hydrolysis observed in the absence of KaiA and KaiB, which is the non-oscillatory condition, is correlated to the modulation of frequency of the phosphorylation rhythm observed in the oscillatory condition^16,17^. The ATPase activity of the truncated CI ring also shows correlation to the oscillation frequency of the phosphorylation rhythm^17^. These observations suggested the intrinsic relation between ATP hydrolysis in the CI and phosphorylation rhythm in the CII^16,18^. In the present paper, we develop a theoretical model to examine the mechanism behind this relation.

Many theoretical models have been proposed to analyze the Kai system^19–27^, but most of them have been devoted to understanding the macroscopic ensemble-level oscillation; by assuming cyclic phosphorylation/dephosphorylation (P/dP) of individual KaiC molecules, various nonlinear mechanisms were proposed to explain how individual oscillations are synchronized to give rise to an ensemble-level oscillation. Example mechanisms were regulation through KaiA sequestration at various phases^8,19–23^ or through monomer shuffling^24–27^. However, the discovery of the correlation between the ATPase activity and the phosphorylation rhythm has indicated that not only the ensemble-level regulation but the regulation within individual KaiC molecules through the interplay between the ATP hydrolysis and P/dP reactions should be important for determining the oscillation features. A detailed molecular-level model of this interplay has been recently developed by Paijmans *et al*.^28^, and the further development of a comprehensive model that bridges between the molecular and ensemble levels is necessary.

## Multifold feedback model

We consider the following binding/unbinding processes among Kai proteins;

1 $$2{\rm{A}}+{{\rm{C}}}_{6}\rightleftharpoons {{\rm{C}}}_{6}{{\rm{A}}}_{2},$$

2 $${\rm{B}}+{{\rm{C}}}_{6}{{\rm{B}}}_{i}{{\rm{A}}}_{2j}\rightleftharpoons {{\rm{C}}}_{6}{{\rm{B}}}_{i+1}{{\rm{A}}}_{2j},\,{\rm{for}}\,0\le i\le 5,\,0\le j\le i,$$

3 $$2{\rm{A}}+{{\rm{C}}}_{6}{{\rm{B}}}_{i}{{\rm{A}}}_{2j}\rightleftharpoons {{\rm{C}}}_{6}{{\rm{B}}}_{i}{{\rm{A}}}_{2j+2},\,{\rm{for}}\,1\le i\le 6,\,0\le j\le i-1.$$

Here, C_6_B_0_A_0_ is identical to C_6_. Probability of the *k*th C_6_ to be bound in forms of C_6_A_2_ and C_6_B_*i*_A_2*j*_ with 0 ≤ *i* ≤ 6 and 0 ≤ *j* ≤ *i* at time *t* are denoted by ${P}_{{{\rm{C}}}_{6}{{\rm{A}}}_{2}}(k,t)$ and ${P}_{{{\rm{C}}}_{6}{{\rm{B}}}_{i}{{\rm{A}}}_{2j}}(k,t)$, respectively with *k* = 1, …, *N*, where *N* is the total copy number of KaiC hexamers in the system. We use the approximation that factorizes an *N*-body probability distribution function into one-body probability distributions, ${P}_{{{\rm{C}}}_{6}{{\rm{A}}}_{2}}(k,t)$ and ${P}_{{{\rm{C}}}_{6}{{\rm{B}}}_{i}{{\rm{A}}}_{2j}}(k,t)$. The similar factorization approximation was used to describe single-molecular fluctuation of binding reactions to DNA^29^. Then, equations for ${P}_{{{\rm{C}}}_{6}{{\rm{A}}}_{2}}(k,t)$ and ${P}_{{{\rm{C}}}_{6}{{\rm{B}}}_{i}{{\rm{A}}}_{2j}}(k,t)$ become

4 $$\frac{d}{dt}{P}_{{{\rm{C}}}_{6}{{\rm{A}}}_{2}}(k,t)={h}_{A}{A}^{2}{P}_{{{\rm{C}}}_{6}{{\rm{B}}}_{0}{{\rm{A}}}_{0}}(k,t)-{f}_{A}{P}_{{{\rm{C}}}_{6}{{\rm{A}}}_{2}}(k,t),$$

5 $$\begin{array}{rcl}\frac{d}{dt}{P}_{{{\rm{C}}}_{6}{{\rm{B}}}_{i}{{\rm{A}}}_{2j}}(k,t) & = & \mathrm{(7}-i){h}_{B}B{P}_{{{\rm{C}}}_{6}{{\rm{B}}}_{i-1}{{\rm{A}}}_{2j}}(k,t)-(i-j){f}_{B}{P}_{{{\rm{C}}}_{6}{{\rm{B}}}_{i}{{\rm{A}}}_{2j}}(k,t)\\  &  & -\mathrm{(6}-i){h}_{B}B{P}_{{{\rm{C}}}_{6}{{\rm{B}}}_{i}{{\rm{A}}}_{2j}}(k,t)+(i+1-j){f}_{B}{P}_{{{\rm{C}}}_{6}{{\rm{B}}}_{i+1}{{\rm{A}}}_{2j}}(k,t)\\  &  & +(i-j+\mathrm{1)}{h}_{BA}{A}^{2}{P}_{{{\rm{C}}}_{6}{{\rm{B}}}_{i}{{\rm{A}}}_{2j-2}}(k,t)-j{f}_{BA}{P}_{{{\rm{C}}}_{6}{{\rm{B}}}_{i}{{\rm{A}}}_{2j}}(k,t)\\  &  & -(i-j){h}_{BA}{A}^{2}{P}_{{{\rm{C}}}_{6}{{\rm{B}}}_{i}{{\rm{A}}}_{2j}}(k,t)+(j+\mathrm{1)}{f}_{BA}{P}_{{{\rm{C}}}_{6}{{\rm{B}}}_{i}{{\rm{A}}}_{2j+2}}(k,t),\end{array}$$

where *A* and *B* are concentrations of unbound free KaiA and KaiB, respectively. See Methods section for more details. In Eq. 5, the rate constants of binding, *h*_*A*_ and *h*_*B*_, and the rates of unbinding, *f*_*A*_ and *f*_*B*_, should depend on the structure of individual KaiC hexamers, but we assume that *h*_*BA*_ or *f*_*BA*_ does not depend on the KaiC structure because KaiA in C_6_B_*i*_A_2*j*_ binds to KaiB and does not directly binds to KaiC. In solving Eqs 4 and 5, we have the constraints,

6 $$\begin{array}{ccc}1 & = & {P}_{{{\rm{C}}}_{6}{{\rm{A}}}_{2}}(k,t)+\sum _{i=0}^{6}\,\sum _{j=0}^{i}\,{P}_{{{\rm{C}}}_{6}{{\rm{B}}}_{i}{{\rm{A}}}_{2j}}(k,t),\,{\rm{f}}{\rm{o}}{\rm{r}}\,k=1,\ldots ,N,\\ {A}_{T} & = & A+\frac{2}{V}\,\sum _{k=1}^{N}\,({P}_{{{\rm{C}}}_{6}{{\rm{A}}}_{2}}(k,t)+\sum _{i=1}^{6}\,\sum _{j=1}^{i}\,j{P}_{{{\rm{C}}}_{6}{{\rm{B}}}_{i}{{\rm{A}}}_{2j}}(k,t)),\\ {B}_{T} & = & B+\frac{1}{V}\,\sum _{k=1}^{N}\,\sum _{i=1}^{6}\,\sum _{j=0}^{i}\,i{P}_{{{\rm{C}}}_{6}{{\rm{B}}}_{i}{{\rm{A}}}_{2j}}(k,t),\end{array}$$

where *V* is the system volume, and *A*_*T*_ and *B*_*T*_ are total concentrations of KaiA and KaiB, respectively.

The small-angle X-ray scattering^30^, the NMR spectroscopy^31,32^, and the biochemical analysis^33^ showed that during the phase of phosphorylation (P), KaiC hexamer takes a structure different from that during the phase of dephosphorylation (dP). Oyama *et al*. referred to the structure in the P-phase as ground state (gs), and the one in the dP-phase as competent state (cs); cs-KaiC can dissociate into monomers and is less stable than gs-KaiC^33^. We assume six subunits in a KaiC hexamer cooperatively undergo the structural transition for the hexamer to exhibit two structural states as assumed in previous models^20,24,25^; we represent this allosteric transition by a continuous variable *X* as *X*_*k*_ ≈ 1 when the *k*th KaiC hexamer is in the gs-state and *X*_*k*_ ≈ 0 when in the cs-state. Then, the rate constants in Eqs 4 and 5 should depend on the structural order parameter *X*_*k*_ as

7 $$\begin{array}{ll}\begin{array}{r}{h}_{A}(k,t)={h}_{A0}{X}_{k},\\ {h}_{B}(k,t)={h}_{B0}(1-{X}_{k}),\end{array} & \begin{array}{l}{f}_{A}(k,t)={f}_{A0}\mathrm{(1}-{X}_{k}),\\ {f}_{B}(k,t)={f}_{B0}{X}_{k},\end{array}\end{array}$$

where *h*_*A*0_, *f*_*A*0_, *h*_*B*0_, and *f*_*B*0_ are constants. With this definition of rate constants, KaiA binds to KaiC with larger affinity in the gs-state, which promotes P-reactions, while KaiB binds to KaiC with larger affinity in the cs-state, which promotes dP-reactions.

In order to facilitate the extensive calculation of the many-molecular system, we adopt a simplified representation of the phosphorylation level of 12 sites in a single KaiC hexamer by using a continuous variable *D* as *D*_*k*_(*t*) ≈ 1 when 12 sites of the *k*th KaiC hexamer are fully phosphorylated, and *D*_*k*_(*t*) ≈ 0 when they are fully dephosphorylated. *D*_*k*_ is increased when KaiA binds to KaiC to form C_6_A_2_ and is decreased otherwise. This transition is represented by the equation of soft-spin dynamics as

8 $$\frac{d{D}_{k}}{dt}={k}_{p}{P}_{{{\rm{C}}}_{6}{{\rm{A}}}_{2}}(k,t)-{k}_{dp}\,(1-{P}_{{{\rm{C}}}_{6}{{\rm{A}}}_{2}}(k,t))-\frac{\partial }{\partial {D}_{k}}g({D}_{k}),$$

where *k*_*p*_ and *k*_*dp*_ are rate constants of P and dP reactions, respectively, and *g*(*D*) = *aD*(*D* − 1/2)^2^(*D* − 1) with *a* > 0 represents a soft-spin constraint to confine *D* in a finite range. We assume that structure transition is much faster than the other reactions, so that the structure *X*_*k*_ is represented as a quasi-equilibrium average under the mean-field generated by *D*_*k*_ and other quantities as

9 $${X}_{k}(t)=\frac{1}{2}\,\tanh \,[\beta ({c}_{0}-{c}_{1}{D}_{k}(t)+{c}_{2}{p}_{k}^{A}(t)-{c}_{3}{p}_{k}^{B}(t)-{q}_{k}(t))]+\frac{1}{2},$$

where *β* = 1/*k*_B_ *T*, and *c*_0_, *c*_1_, *c*_2_, and *c*_3_ are constants. ${p}_{k}^{A}(t)={P}_{{{\rm{C}}}_{6}{{\rm{A}}}_{2}}(k,t)$ and ${p}_{k}^{B}(t)={\sum }_{i=1}^{6}\,\tanh (i/{n}_{B})\,{\sum }_{j=0}^{i}\,{P}_{{{\rm{C}}}_{6}{{\rm{B}}}_{i}{{\rm{A}}}_{2j}}$ $(k,t)$ represent the extent of KaiA binding and that of KaiB binding, respectively, where *n*_*B*_ represents the level of saturation of the effects of KaiB binding. See Methods section. *q*_*k*_ represents the effect of ATPase reactions as explained later.

Equations 4–9 constitute loops of feedback among P/dP reactions and structural transitions, which brings about nonlinear behaviors of *X*_*k*_(*t*) and *D*_*k*_(*t*). When *c*_3_ > 0 in Eq. 9, for example, the larger probability of KaiB binding to KaiC brings the structure to the cs-state with a small *X*_*k*_, which further enhances the KaiB binding through Eqs 5 and 7. Therefore, the *c*_3_ > 0 term gives a positive feedback effect. If we would neglect the effects of the *c*_1_ term, this positive feedback should stabilize the (small *X*_*k*_, small *D*_*k*_) state for ${p}_{k}^{B}\approx 1$ and the (large *X*_*k*_, large *D*_*k*_) state for ${p}_{k}^{B}\approx 0$. On the other hand, when *c*_1_ > 0 in Eq. 9, the large *D*_*k*_ leads the KaiC structure to the cs-state with small *X*_*k*_. From Eq. 7, this brings about the small affinity of KaiA to KaiC, reducing ${P}_{{{\rm{C}}}_{6}{{\rm{A}}}_{2}}(k,t)$ in Eq. 4, which in turn reduces *D*_*k*_ in Eq. 8. Therefore, the *c*_1_ > 0 term gives a negative feedback effect, which should stabilize the state with (large *X*_*k*_, small *D*_*k*_) or (small *X*_*k*_, large *D*_*k*_). The system tends to stay at these various states, but because of the competition among multiple feedback interactions, the system is easily moved when small perturbations are added to generate oscillation among the states. The *q*_*k*_ term representing the effect of ATPase reactions provides such a perturbation.

The ATPase reactions are dominated by the CI ring^17^, whereas the P/dP reactions take place in the CII ring; therefore, a coordinated structural change of the CI and CII domains, i.e., allosteric communication between CI and CII mediates the effects of ATPase reactions and P/dP reactions. In the present model, *X*_*k*_ represents such communication. There are accumulating experimental data suggesting that the ATPase activity in the CI is necessary for the binding of KaiC to KaiB^31,33,34^, indicating that the ATP hydrolysis enhances the binding affinity of KaiC to KaiB. Therefore, a plausible assumption is that the ATP hydrolysis changes the KaiC structure from gs to cs. This effect is represented in Eq. 9 by setting ${q}_{k}(t)={\sum }_{i=1}^{6}\,q(i;k,t)$ with *q*(*i*; *k*, *t*) = *q*_0_ > 0 for *t*_0_ ≤ *t* ≤ *t*_0_ + *δ*_*k*_ when P_i_ is released at time *t* = *t*_0_ and ADP is kept bound on the CI of the *i*th subunit of *k*th KaiC hexamer for the time duration *δ*_*k*_. KaiC hexamer is prevented from disassembling into monomers when ATP is bound on the CI^5,6^, which suggests *q*(*i*; *k*, *t*) < *q*_0_ when ATP or ADP + P_i_ is bound. Here, we use a simple assumption that *q*(*i*; *k*, *t*) = 0 when ATP or ADP + P_i_ is bound or no nucleotide is bound on the CI of the *i*th subunit. In this model, the P_i_ release is assumed to take place randomly with a frequency *f*_0_ at individual subunits, and the lifetime of the ADP-bound state *δ*_*k*_ in a subunit of the *k*th hexamer is assumed to depend on the structure as

10 $${\delta }_{k}={\delta }_{0}(\gamma -{X}_{k}),$$

where *γ* ≥ 1 is a constant. With the definition of Eq. 10, the ATPase activity does not directly depend on the phosphorylation level or the protein binding state. However, through the structure modulation *X*_*k*_, the ATPase activity is indirectly affected by the phosphorylation level and the protein binding state. The ADP release is more frequent with shorter *δ*_*k*_ in the gs state with larger *X*_*k*_; therefore, ATP is hydrolyzed more frequently during the P-phase as observed in the experiment^16^.

Parameters were chosen so as to make the reactions slow enough; the time scales of P/dP reactions, ${k}_{p}^{-1}$ and ${k}_{dp}^{-1}$, and the time scales of ATPase reactions, ${f}_{0}^{-1}$ and *δ*_0_, were chosen to be of the order of 0.5–1 hour. This estimated order of the slow rates is consistent with the large activation free-energy for those reactions suggested by the structural analyses^17^. The rate of KaiA binding, which is ≈${h}_{A}{A}_{T}^{2}$, was chosen to be fast with the order of minutes to show the strong affinity when *X* ≈ 1, but the rate of KaiB binding, ≈*h*_*B*_ *B*_*T*_, was chosen to be slow with time scale of hours to achieve balance between KaiA binding on a single site of the CII ring and KaiB binding on multiple sites of the CI ring; the slowness of KaiB binding is consistent with the experimental observation^22,35^. Within this estimated order of parameter range, parameters were calibrated to show a stable coherent oscillation of the ensemble average of *D*, $\overline{D}(t)=\tfrac{1}{N}\,{\sum }_{k=1}^{N}\,{D}_{k}(t)$.

## Results

**Figure 2:**
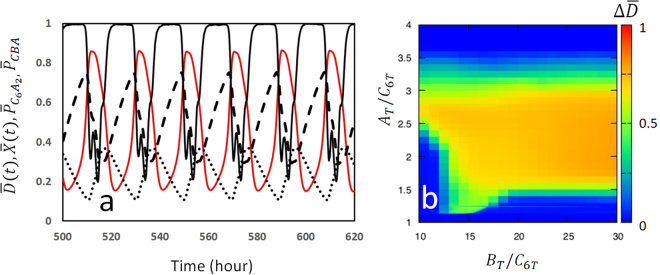
Simulated oscillation of the Kai system. (**a**) The simulated trajectory is shown by plotting averages over the ensemble of KaiC molecules as functions of time; the level of phosphorylation $\overline{D}(t)$ (red), structure $\overline{X}(t)$ (black, real line), probability to form the C_6_A_2_ complex, ${\overline{P}}_{{{\rm{C}}}_{6}{{\rm{A}}}_{2}}$ (dashed line), and probability to form the C_6_B_*i*_A_2*j*_ complexes, ${\overline{P}}_{{\rm{CBA}}}$ (dotted line) are plotted with *A*_*T*_/*C*_6*T*_ = 2 and *B*_*T*_/*C*_6*T*_ = 20, where *C*_6*T*_ = *N*/*V* is the concentration of KaiC hexamer. (**b**) The amplitude of oscillation, ${\rm{\Delta }}\overline{D}$, is plotted on the plane of *A*_*T*_/*C*_6*T*_ and *B*_*T*_/*C*_6*T*_. Using units of *V* = 1 and *k*_B_ *T* = 1, parameters are *N* = 1000, *a* = 1.6 h^−1^, *k*_*p*_ = 0.4 h^−1^, *k*_*dp*_ = 0.4 h^−1^, *c*_0_ = 10, *c*_1_ = 6, *c*_2_ = 0, *c*_3_ = 3, *f*_0_ = 2.4 h^−1^, *q*_0_ = 0.8, *δ*_0_ = 2.5 h, *γ* = 1.5, *n*_*B*_ = 1, *h*_*A*0_ = 1.6 × 10^−5^ h^−1^, *h*_*B*0_ = 7.2 × 10^−6^ h^−1^, *h*_*BA*_ = 1.0 × 10^−6^ h^−1^,  *f*_*A*0_ = 0.8 h^−1^,  *f*_*B*0_ = 0.32 h^−1^, and *f*_*BA*_ = 0.2 h^−1^.

Figure 2a shows an example trajectory obtained by numerically solving Eqs 4–10 for an ensemble of *N* = 1000 KaiC hexamers. Here, the ensemble averages $\overline{D}(t)$ and $\overline{X}(t)=\tfrac{1}{N}\,{\sum }_{k=1}^{N}\,{X}_{k}(t)$ are plotted as functions of time, together with the ensemble average of probabilities ${\overline{P}}_{{{\rm{C}}}_{6}{{\rm{A}}}_{2}}(t)=\tfrac{1}{N}\,{\sum }_{k=1}^{N}\,{P}_{{{\rm{C}}}_{6}{{\rm{A}}}_{2}}(k,t)$ and ${\overline{P}}_{{\rm{CBA}}}(t)=\tfrac{1}{N}\,{\sum }_{k=1}^{N}\,{\sum }_{i=1}^{6}\,{\sum }_{j=1}^{i}\,{P}_{{{\rm{C}}}_{6}{{\rm{B}}}_{i}{{\rm{A}}}_{2j}}(k,t)$. We found that ${\overline{P}}_{{{\rm{C}}}_{6}{{\rm{A}}}_{2}}$ and ${\overline{P}}_{{\rm{CBA}}}$ oscillate in opposite phases, showing that there is a competition between two types of complexes C_6_A_2_ and C_6_B_*i*_A_2*j*_; when C_6_A_2_ dominates, *X* decreases and *D* increases with the action of KaiA on KaiC hexamers, and when C_6_B_*i*_A_2*j*_ dominates, there is not sufficient KaiA to form C_6_A_2_, which decreases *D* and increases *X*. In the latter case, KaiA is sequestered in C_6_B_*i*_A_2*j*_, which is the mechanism of synchronization of many KaiC hexamers and is necessary for the ensemble-level oscillation. This effect is shown in Fig. 2b, where the amplitude of $\overline{D}$ oscillation, ${\rm{\Delta }}\overline{D}$, is plotted. ${\rm{\Delta }}\overline{D}$ is large when the KaiA concentration is larger than a threshold of *A*_*T*_/*C*_6*T*_ ≈ 1.4, but with too large KaiA concentration, the sequestration mechanism does not work and the synchronization is lost to make ${\rm{\Delta }}\overline{D}\approx 0$. On the other hand, the upper limit of the KaiB concentration for the proper oscillation was not found within the reasonable range because the sequestration mechanism is not working for KaiB. Existence of such concentration ranges of KaiA and KaiB agrees with the experimental observation^36^ and is similar to the results of the previous Monte Carlo-type simulation^20^ in which sequestration of KaiA at a different oscillatory phase from the present model was assumed.

**Figure 3:**
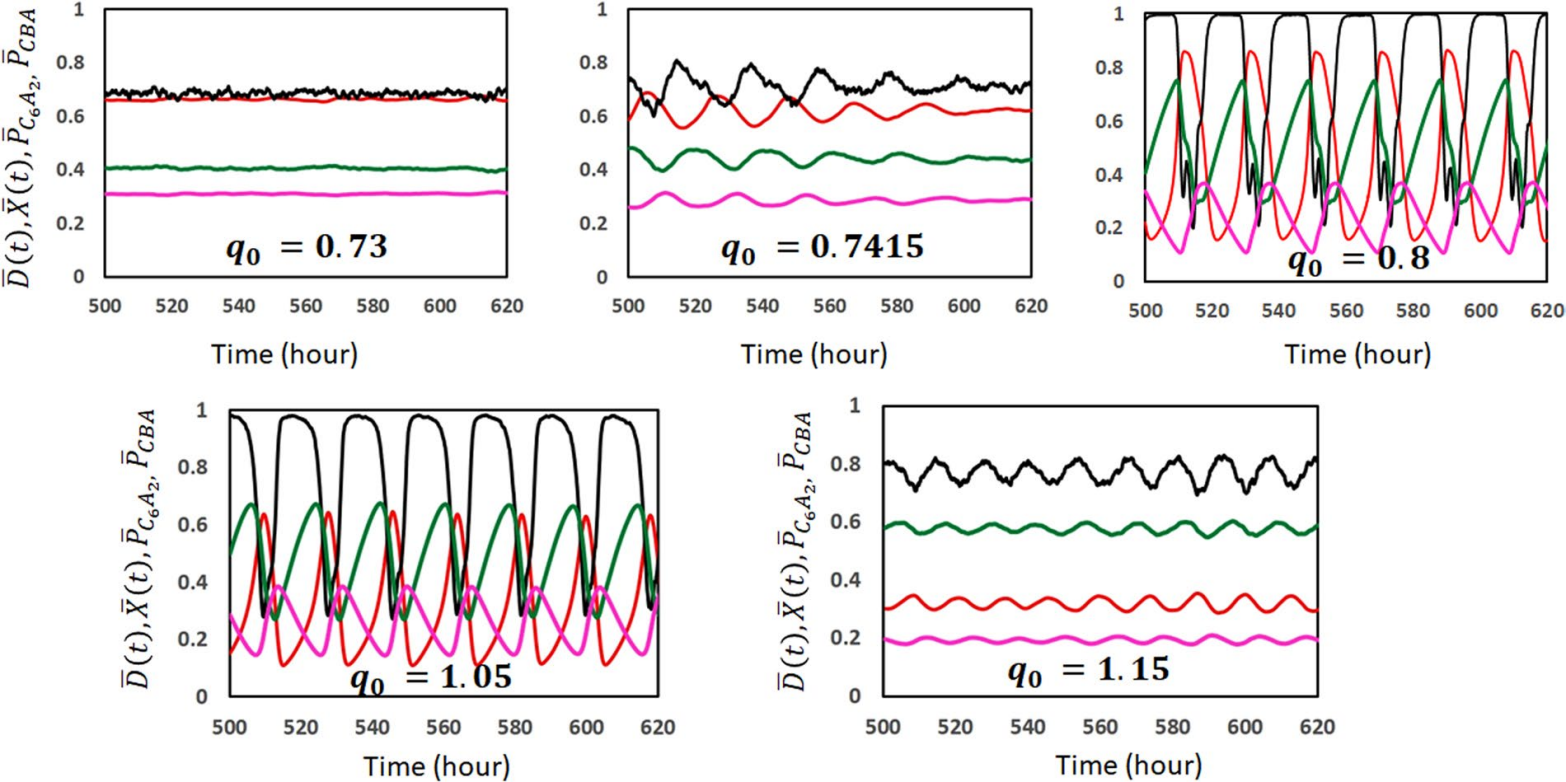
Example trajectories calculated with different values of *q*_0_. The ensemble averages of KaiC phosphorylation level $\overline{D}$ (red), KaiC hexamer structure $\overline{X}$ (black), probability to form the C_6_A_2_ complex, ${\overline{P}}_{{{\rm{C}}}_{6}{{\rm{A}}}_{2}}$ (green), and probability to form the C_6_B_*i*_A_2*j*_ complexes, ${\overline{P}}_{{\rm{CBA}}}$ (pink) are plotted as functions of time. The value of *q*_0_ is written in units of *k*_B_ *T* in each panel. Parameters other than *q*_0_ are the same as explained in the caption of Fig. 2.

**Figure 4:**
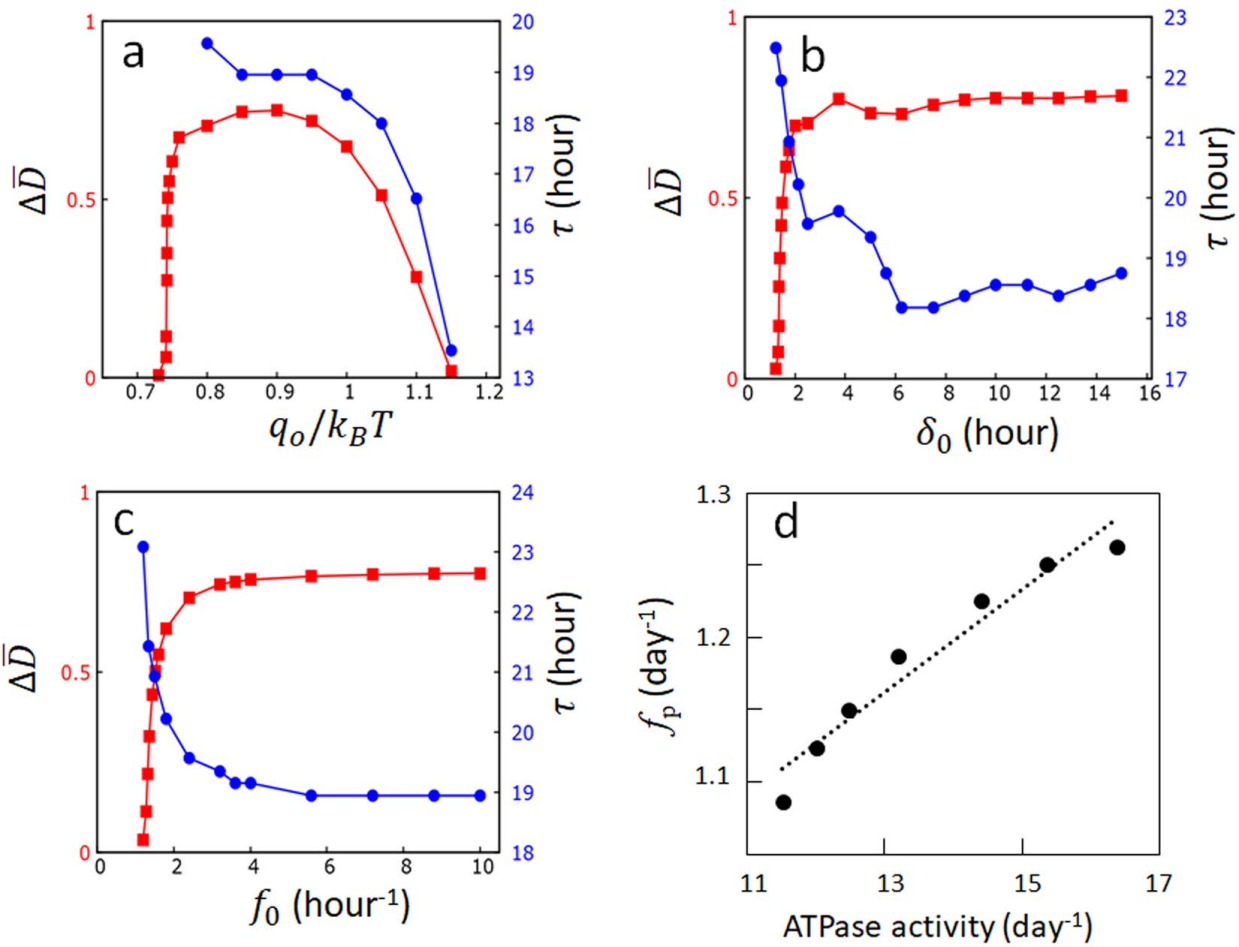
Effects of ATPase reactions on the circadian oscillation of the Kai system. (**a**–**c**) The oscillation amplitude ${\rm{\Delta }}\overline{D}$ (red) and the oscillation period *τ* (blue) are plotted as functions of (**a**) the amplitude of impact of the ADP bound state on the structure, *q*_0_, (**b**) the lifetime of the ADP bound state, *δ*_0_, and (**c**) the frequency of the Pi release, *f*_0_. (**d**) The correlation between ATPase activity and oscillation frequency is shown by simulating with various values of *f*_0_, where the ATPase activity was calculated in the condition of *A*_*T*_ = *B*_*T*_ = 0 and the oscillation frequency, *f*_p_, was calculated with *A*_*T*_/*C*_6*T*_ = 2 and *B*_*T*_/*C*_6*T*_ = 20. Parameters used are the same as in Fig. 2 except for those varied in each graph. Each point is the average of the data calculated from 10 trajectories, each having 2 × 10^3^ h length.

Effects of ATPase reactions are analyzed by changing *q*_0_, *δ*_0_, and *f*_0_, which are the parameters to define the ATPase reactions in the model. Figure 3 shows how the simulated trajectory changes when the degree of influence of ATPase reaction to the structure, *q*_0_, is changed. When *q*_0_ is too small, the ATPase reactions are insufficient for perturbing the system from the steady state with large $\overline{X}$ and large $\overline{D}$. However, when *q*_0_ is too large, the ATPase reactions stabilize the (large $\overline{X}$, small $\overline{D}$) state and prevents oscillation. This is summarized in Fig. 4a by plotting the oscillation amplitude, ${\rm{\Delta }}\overline{D}$, which becomes large when *q*_0_ exceeds a certain threshold; therefore, ${\rm{\Delta }}\overline{D}\ne 0$ only within a limited range of *q*_0_. Also plotted in Fig. 4a is the oscillation period *τ*, which is defined by *τ* = 1/*f*_p_ with *f*_p_ being the frequency of the peak of Fourier transformed spectrum of $\overline{D}(t)$; *τ* is a decreasing function of *q*_0_, showing that the small and quick oscillation around the (large $\overline{X}$, small $\overline{D}$) state dominates when *q*_0_ is large. Figure 4b shows that ${\rm{\Delta }}\overline{D}\ne 0$ when the lifetime of ADP bound state, *δ*_0_, exceeds a certain threshold. For the large enough *δ*_0_ with ${\delta }_{0}{f}_{0}\gg 1$, the CI of each subunit almost always binds ADP and the system behavior shows saturation. *τ* shows a complex behavior as *δ*_0_ is varied because the form of oscillation as a function of time is varied by changing *δ*_0_. The similar threshold and saturation behaviors are found in Fig. 4c when the frequency of ATP hydrolysis, *f*_0_, is varied.

Thus, the steady state turns into oscillation when the quantities that define the ATPase reactions in the model, *q*_0_, *δ*_0_, and *f*_0_, are larger than certain thresholds. Therefore, in the present model, ATP hydrolysis, which is randomly processed in individual CI domains, is a driving mechanism of the ensemble-level oscillation.

Notably, the oscillation period is modulated by changes in *q*_0_, *δ*_0_, and *f*_0_. Among them, the frequency of ATP hydrolysis, or the ATPase activity of KaiC, is most sensitively dependent on *f*_0_. In Fig. 4d, the averaged number of released ADP from each CI in 24 h is calculated as a measure of the ATPase activity in the non-oscillatory condition of *A*_*T*_ = *B*_*T*_ = 0 for various values of *f*_0_, and the thus calculated ATPase activity is compared with the frequency *f*_p_ of phosphorylation rhythm calculated in the oscillatory condition. We find a clear correlation between ATPase activity and *f*_p_, and the slope of the line fitted to the results in Fig. 4d is 0.036. Because it is plausible that *f*_0_ should be modulated by mutations, the calculated slope is consistent with the observed slope of 0.03–0.04^17^ and 0.055^16^ obtained from various mutant data. Thus, the perturbation of structure by the ATPase reactions in individual molecules is a key determinant of the oscillation frequency in the ensemble of many molecules.

**Figure 5:**
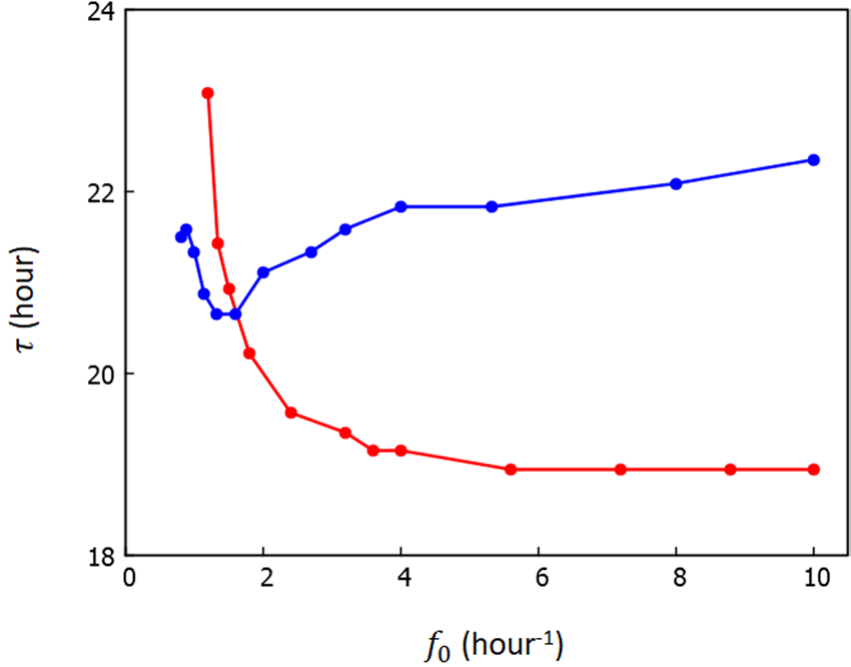
The oscillation period *τ* is calculated by varying *f*_0_. Change of *τ* is much smaller when *f*_0_ *δ*_0_ is kept constant as *f*_0_ *δ*_0_ = 4 (blue) than when *δ*_0_ is kept constant as *δ*_0_ = 2.5 h (red). Other parameters are the same as explained in the caption of Fig. 2. Each point is the average of the data calculated from 10 trajectories, each having 2 × 10^3^ h length.

As temperature is increased, reactions should be accelerated, which should increase *f*_0_ and decrease *δ*_0_. It is suggestive that in Fig. 4b,c, the oscillation period is shortened when *f*_0_ is increased and prolonged when *δ*_0_ is decreased, so that the period is not changed largely when *f*_0_ *δ*_0_ is kept constant as shown in Fig. 5. Note that *τ* only insensitively depends on *f*_0_ and *δ*_0_ when *f*_0_ and *δ*_0_ are large enough as shown in Fig. 4; therefore, the oscillation period becomes insensitive to temperature change when *f*_0_ or *δ*_0_ is regulated to be large enough even in the case *f*_0_ *δ*_0_ is not kept constant. Further careful analyses are needed to elucidate the temperature dependence of *f*_0_ and *δ*_0_, but the results in the present model showed that the control of oscillation period with ATPase reactions may work as a mechanism for the observed insensitivity of the oscillation period to temperature, i.e., temperature compensation, together with the ensemble-level mechanism of regulation for temperature compensation^21^.

## Discussion

**Figure 6:**
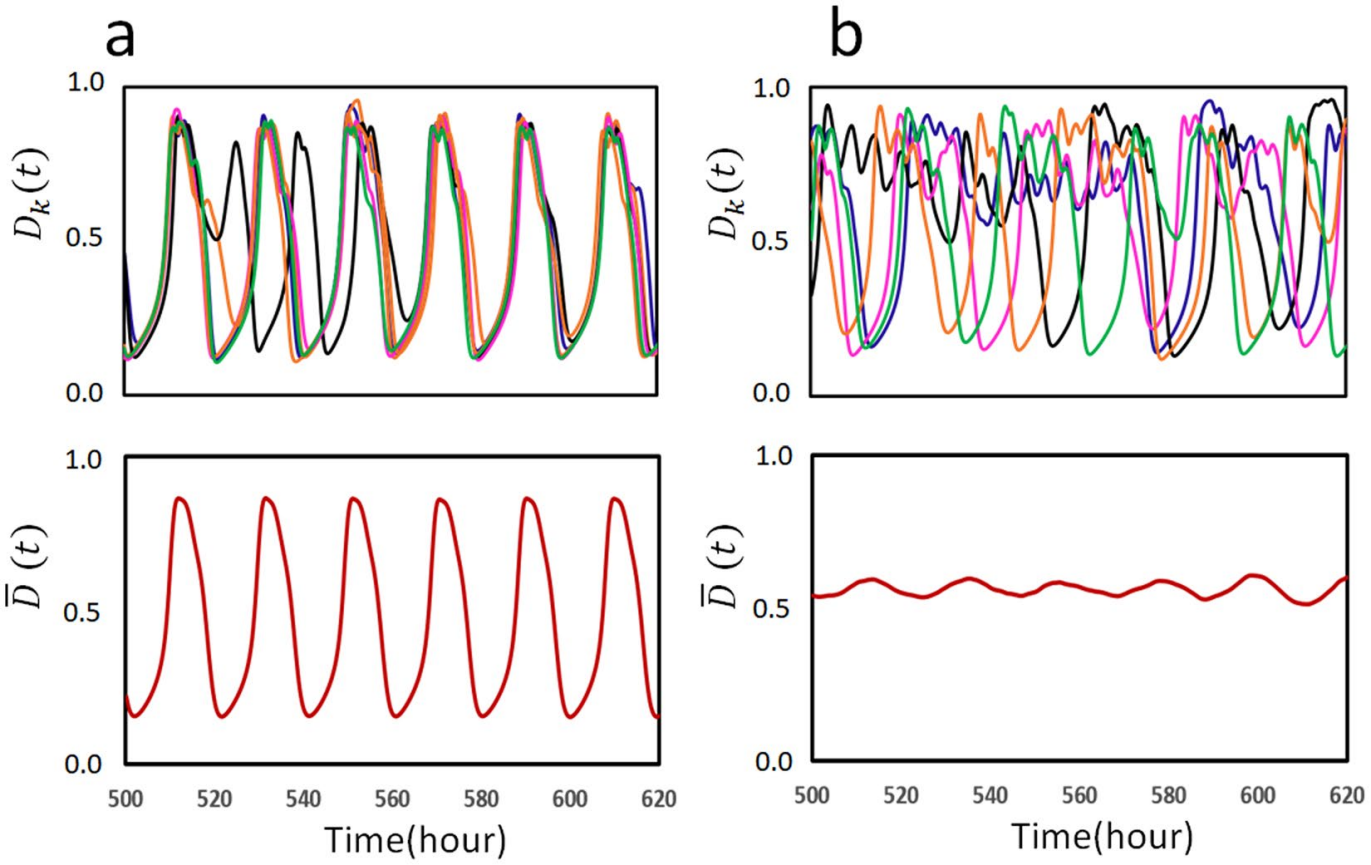
Stochastic fluctuation of oscillation of individual KaiC hexamers. Frequency of the P_i_ release, *f*_0_, is (**a**) *f*_0_ = 2.4 h^−1^ and (**b**) *f*_0_ = 1.2 h^−1^. In top panels, simulated oscillations, *D*_*k*_(*t*), of five KaiC hexamers are superposed; these hexamers were arbitrarily chosen from the ensemble of *N* = 1000 hexamers. Oscillations of different hexamers are shown with different colors. In bottom panels, the simulated oscillation of the ensemble, $\overline{D}(t)$, is shown. Parameter values other than *f*_0_ are the same as explained in the caption of Fig. 2.

It is interesting to see how the stochastic fluctuation of oscillation is regulated by the ATP hydrolysis reactions. In the present model, due to the stochastic timing of ATP hydrolysis reactions in each CI domain of individual KaiC hexamers, the simulated oscillation of individual KaiC hexamers, *D*_*k*_, shows stochastic fluctuation even in the case that the ensemble average $\overline{D}(t)$ shows a coherent oscillation. In Fig. 6, the stochastic fluctuation of individual KaiC hexamers is shown by superposing *D*_*k*_(*t*) of several example KaiC hexamers, which were arbitrarily chosen from the *N*-hexamer ensemble. When the ATPase frequency, *f*_0_, is large enough, individual oscillatory trajectories *D*_*k*_(*t*) are fluctuating around the coherent oscillation $\overline{D}(t)$ of the ensemble average (Fig. 6a). When *f*_0_ is too small, on the other hand, synchronization among individual oscillations becomes weak and the amplitude of the ensemble average oscillation $\overline{D}$ becomes small (Fig. 6b). Therefore, the ATP hydrolysis reactions are necessary for synchronizing individual stochastic oscillations to give rise to a coherent ensemble-level oscillation.

The present analyses showed that individual stochastic oscillations are synchronized through sequestration of KaiA into C_6_B_*i*_A_2*j*_, and the synchronization promoted by this sequestration is enhanced by the frequent ATPase reactions; the frequent structural modulation induced by the ATPase reactions should perturb individual molecules, and this perturbation is necessary for individual hexamers to adjust to the ensemble-level oscillation. It is necessary to further analyze this mechanism quantitatively by comparing the degree of synchronization and the free energy flow induced by the ATP consumption. Theoretical description with combined use of landscape and probability-flux representations^23,37^ should provide a useful means for this analysis.

We developed a simplified model of the reconstituted circadian clock. In particular, it was assumed that the binding affinity of KaiA and KaiB to KaiC depends on the structure *X*_*k*_(*t*) of KaiC hexamer as defined in Eq. 7. In contrast to the assumption of phosphorylation-level dependent binding in many of the previous theoretical models^8,19–21,23,25,28^, the direct explicit dependence of the binding affinity on the phosphorylation level *D*_*k*_(*t*) of KaiC was not considered in the present model. Instead, it was assumed that the phosphorylation level *D*_*k*_(*t*) only indirectly affects the binding affinity through the modulation of the phosphorylation-level dependent structure *X*_*k*_(*t*) as shown in Eqs 7 and 9. This assumption should be reasonable because the atomic positions of the phosphorylation sites, Ser431 and Thr432, are distant from the binding sites of KaiA^11^ and KaiB^13^, so that the allosteric communication through the change in *X*_*k*_(*t*) should mediate the effects of P/dP reactions and binding of KaiA or KaiB. In a similar way, the ATPase activity was assumed to depend on *X*_*k*_(*t*) as shown in Eq. 10 by neglecting the direct dependence of the ATPase activity on *D*_*k*_(*t*); the ATPase activity is only indirectly affected by *D*_*k*_(*t*) through Eqs 7 and 10. Thus, in the present model, different types of reactions, P/dP reactions, ATP hydrolysis, and Kai protein binding reactions, induce the allosteric structural transition, and the allosteric structural transition regulates these reactions, which constitutes multifold feedback interactions among reactions and structural transition. In the present paper, we focused on the intra-molecular dynamics induced by these multifold feedback interactions and analyzed the correlation between intra-molecular dynamics and the inter-molecular dynamics which gives rise to the ensemble-level oscillation. The model has rooms for improvement in explaining the further detailed features of the system. For instance, two sites of phosphorylation should be distinguished by using variables for individual phosphorylation sites, and structures of CI and CII in each subunit should be described by using structural variables for individual domains. Temperature dependence of each parameter should be carefully examined to analyze the mechanism of temperature compensation. Extension of the present model to these directions is an important avenue for further research.

The Kai oscillator provides a clear example that the intra-molecular regulation through the feedback relations among reactions and structure and the inter-molecular system-level regulation through concentration change are coupled with each other to realize an integrated behavior of stable oscillation. Physical perspective obtained by the analyses of this coupling should give further insights on the multilevel aspects of this important system.

## Methods

### Kinetic equations for the Kai protein binding

For the system having *N* hexamers of KaiC, stochastic dynamics of the system should be described by the master equation, which is the kinetic differential equation for *P*(*n*_1_, *i*_1_, *j*_1_; *n*_2_, *i*_2_, *j*_2_; …; *n*_*N*_, *i*_*N*_, *j*_*N*_), where *n*_*k*_ = *i*_*k*_ = *j*_*k*_ = 0 when KaiA or KaiB is not bound to the *k*th KaiC and *n*_*k*_ = 1 and *i*_*k*_ = *j*_*k*_ = 0 when a KaiA dimer binds to the CII ring of the *k*th KaiC. *i*_*k*_ is the number of bound KaiB on the CI ring of the *k*th KaiC and *j*_*k*_ is the number of bound KaiA dimers on KaiB. Eqs 4 and 5 can be derived by using the Hartree-like approximation^29^ as $P({n}_{1},{i}_{1},{j}_{1};{n}_{2},{i}_{2},{j}_{2};\ldots ;{n}_{N},{i}_{N},{j}_{N})={\prod }_{k=1}^{N}\,P({n}_{k},{i}_{k},{j}_{k})$ and writing ${P}_{{{\rm{C}}}_{6}{{\rm{A}}}_{2}}(k,t)=P({n}_{k}=1,0,\mathrm{0)}$, and ${P}_{{{\rm{C}}}_{6}{{\rm{B}}}_{i}{{\rm{A}}}_{2j}}(k,t)=P\mathrm{(0},{i}_{k}=i,{j}_{k}=j)$.

We note that Eq. 5 is the expression for the case of 2 ≤ *i* ≤ 5 and 1 ≤ *j* ≤ *i* − 1. For the other values of *i* or *j*, the corresponding equations are

11 $$\begin{array}{rcl}\frac{d}{dt}{P}_{{{\rm{C}}}_{6}{{\rm{B}}}_{0}{{\rm{A}}}_{0}}(k,t) & = & -{h}_{A}{A}^{2}{P}_{{{\rm{C}}}_{6}{{\rm{B}}}_{0}{{\rm{A}}}_{0}}(k,t)+{f}_{A}{P}_{{{\rm{C}}}_{6}{{\rm{A}}}_{2}}(k,t)\\  &  & -6{h}_{B}B{P}_{{{\rm{C}}}_{6}{{\rm{B}}}_{0}{{\rm{A}}}_{0}}(k,t)+{f}_{B}{P}_{{{\rm{C}}}_{6}{{\rm{B}}}_{1}{{\rm{A}}}_{0}}(k,t),\end{array}$$

12 $$\begin{array}{rcl}\frac{d}{dt}{P}_{{{\rm{C}}}_{6}{{\rm{B}}}_{i}{{\rm{A}}}_{0}}(k,t) & = & \mathrm{(7}-i){h}_{B}B{P}_{{{\rm{C}}}_{6}{{\rm{B}}}_{i-1}{{\rm{A}}}_{0}}(k,t)-i{f}_{B}{P}_{{{\rm{C}}}_{6}{{\rm{B}}}_{i}{{\rm{A}}}_{0}}(k,t)\\  &  & -\mathrm{(6}-i){h}_{B}B{P}_{{{\rm{C}}}_{6}{{\rm{B}}}_{i}{{\rm{A}}}_{0}}(k,t)+(i+\mathrm{1)}{f}_{B}{P}_{{{\rm{C}}}_{6}{{\rm{B}}}_{i+1}{{\rm{A}}}_{0}}(k,t)\\  &  & -i{h}_{BA}{A}^{2}{P}_{{{\rm{C}}}_{6}{{\rm{B}}}_{i}{{\rm{A}}}_{0}}(k,t)+{f}_{BA}{P}_{{{\rm{C}}}_{6}{{\rm{B}}}_{i}{{\rm{A}}}_{2}}(k,t),\,{\rm{for}}\,1\le i\le 5\end{array}$$

13 $$\begin{array}{rcl}\frac{d}{dt}{P}_{{{\rm{C}}}_{6}{{\rm{B}}}_{i}{{\rm{A}}}_{2i}}(k,t) & = & -\mathrm{(6}-i){h}_{B}B{P}_{{{\rm{C}}}_{6}{{\rm{B}}}_{i}{{\rm{A}}}_{2i}}(k,t)+{f}_{B}{P}_{{{\rm{C}}}_{6}{{\rm{B}}}_{i+1}{{\rm{A}}}_{2i}}(k,t)\\  &  & +{h}_{BA}{A}^{2}{P}_{{{\rm{C}}}_{6}{{\rm{B}}}_{i}{{\rm{A}}}_{\mathrm{2(}i-\mathrm{1)}}}(k,t)-i{f}_{BA}{P}_{{{\rm{C}}}_{6}{{\rm{B}}}_{i}{{\rm{A}}}_{2i}}(k,t),\,{\rm{for}}\,1\le i\le 5,\end{array}$$

14 $$\begin{array}{rcl}\frac{d}{dt}{P}_{{{\rm{C}}}_{6}{{\rm{B}}}_{6}{{\rm{A}}}_{2j}}(k,t) & = & {h}_{B}B{P}_{{{\rm{C}}}_{6}{{\rm{B}}}_{5}{{\rm{A}}}_{j}}(k,t)-\mathrm{(6}-j){f}_{B}{P}_{{{\rm{C}}}_{6}{{\rm{B}}}_{6}{{\rm{A}}}_{2j}}(k,t)\\  &  & +\mathrm{(7}-j){h}_{BA}{A}^{2}{P}_{{{\rm{C}}}_{6}{{\rm{B}}}_{6}{{\rm{A}}}_{\mathrm{2(}j-\mathrm{1)}}}(k,t)-j{f}_{BA}{P}_{{{\rm{C}}}_{6}{{\rm{B}}}_{6}{{\rm{A}}}_{2j}}(k,t),\\  &  & -\mathrm{(6}-j){h}_{BA}{A}^{2}{P}_{{{\rm{C}}}_{6}{{\rm{B}}}_{6}{{\rm{A}}}_{2j}}(k,t)+(j+\mathrm{1)}{f}_{BA}{P}_{{{\rm{C}}}_{6}{{\rm{B}}}_{6}{{\rm{A}}}_{\mathrm{2(}j+\mathrm{1)}}}(k,t),\,{\rm{for}}\,1\le j\le 5,\end{array}$$

15 $$\begin{array}{rcl}\frac{d}{dt}{P}_{{{\rm{C}}}_{6}{{\rm{B}}}_{6}{{\rm{A}}}_{0}}(k,t) & = & {h}_{B}B{P}_{{{\rm{C}}}_{6}{{\rm{B}}}_{5}{{\rm{A}}}_{0}}(k,t)-6{f}_{B}{P}_{{{\rm{C}}}_{6}{{\rm{B}}}_{6}{{\rm{A}}}_{0}}(k,t)\\  &  & -6{h}_{BA}{A}^{2}{P}_{{{\rm{C}}}_{6}{{\rm{B}}}_{6}{{\rm{A}}}_{0}}(k,t)+{f}_{BA}{P}_{{{\rm{C}}}_{6}{{\rm{B}}}_{6}{{\rm{A}}}_{2}}(k,t),\end{array}$$

16 $$\frac{d}{dt}{P}_{{{\rm{C}}}_{6}{{\rm{B}}}_{6}{{\rm{A}}}_{12}}(k,t)={h}_{BA}{A}^{2}{P}_{{{\rm{C}}}_{6}{{\rm{B}}}_{6}{{\rm{A}}}_{10}}(k,t)-6{f}_{BA}{P}_{{{\rm{C}}}_{6}{{\rm{B}}}_{6}{{\rm{A}}}_{12}}(k,t\mathrm{)}.$$

### Mean-field equation for the structural transition

The mean-field relation of Eq. 9 is derived by assuming that free energy *G*_*k*_ of the *k*th KaiC hexamer is represented as a function of the structural order parameter of the *i*th subunit, *x*_*i*_(*k*), as

17 $$\begin{array}{rcl}{G}_{k}(\{{x}_{i}(k)\}) & = & -\frac{1}{6}\,\sum _{i=1}^{6}\,{x}_{i}(k)\,({c}_{0}-{c}_{1}{D}_{k}+{c}_{2}{p}_{k}^{A}-{c}_{3}{p}_{k}^{B}-{q}_{k})\\  &  & -J\,(\sum _{i=1}^{5}\,{x}_{i}(k){x}_{i+1}(k)+{x}_{6}(k){x}_{1}(k)),\end{array}$$

where *x*_*i*_(*k*) is an Ising-like variavle with *x*_*i*_(*k*) = 1 when the *i*th subunit is in the gs state and *x*_*i*_(*k*) = −1 when it is in the cs state. When the structure transition is fast enough compared to the other reactions, variables *D*_*k*_, ${p}_{k}^{A}$, ${p}_{k}^{B}$, and *q*_*k*_ can be regarded as static fields acting on *x*_*i*_(*k*). *J* is the strength of a coupling between neighboring subunits, representing the cooperativity of the allosteric structural transition. In the case that $J\gg {k}_{{\rm{B}}}T$, we can regard *x*_*i*_(*k*) does not depend on *i* as *x*_*i*_(*k*) = *x*(*k*). Then, by regarding ${G}_{k}=-x(k)\,({c}_{0}-{c}_{1}{D}_{k}+{c}_{2}{p}_{k}^{A}-{c}_{3}{p}_{k}^{B}-{q}_{k})$ as an Ising-spin Hamiltonian, the quasi-equilibrium average of *x*(*k*) leads to

18 $$\langle x(k)\rangle =\,\tanh \,[\beta ({c}_{0}-{c}_{1}{D}_{k}+{c}_{2}{p}_{k}^{A}-{c}_{3}{p}_{k}^{B}-{q}_{k})].$$

By defining *X*_*k*_ as *X*_*k*_ = (〈*x*(*k*)〉 + 1)/2 and regarding *D*_*k*_, ${p}_{k}^{A}$, ${p}_{k}^{B}$, and *q*_*k*_ as slowly changing time-dependent variables, Eq. 18 becomes Eq. 9. It is an intriguing issue to examine whether the free energy representation of Eq. 17 is valid by using atomistic molecular dynamics simulation.
